# Harnessing Nanozyme Catalytic Activities for Multi-Target Intervention in Neurodegenerative Pathology

**DOI:** 10.3390/nano16211355

**Published:** 2026-10-25

**Authors:** Yanan Wang, Jintao Cheng, Tao Liu, Shiyu Li, Qingchi Wang, Kan Shao, Guiling Yang, Peiwu Li

**Affiliations:** 1 Xianghu Laboratory, Zhejiang Provincial Laboratory of Agriculture, Biomanufacturing Research Institute, Hangzhou 311231, China; wangyanan_cau@126.com (Y.W.); jintaocheng@zju.edu.cn (J.C.); liutao@xhlab.ac.cn (T.L.); lishiyu@xhlab.ac.cn (S.L.); wangqingchi@xhlab.ac.cn (Q.W.); kshao@iu.edu (K.S.); 2 Oil Crops Research Institute, Chinese Academy of Agricultural Sciences, Wuhan 430062, China; 3 Department of Environmental and Occupational Health, School of Public Health, Indiana University, Bloomington, IN 47405, USA; 4 State Key Laboratory for Managing Biotic and Chemical Threats to the Quality and Safety of Agro-Products, Laboratory (Hangzhou) for Risk Assessment of Agricultural Products of Ministry of Agriculture, Institute of Agro-Product Safety and Nutrition, Zhejiang Academy of Agricultural Sciences, Hangzhou 310021, China

**Keywords:** neurodegenerative diseases, nanozyme, neuroinflammation, oxidative stress, protein aggregation, blood–brain barrier

## Abstract

The therapeutic landscape for neurodegenerative diseases (NDs), including Alzheimer’s and Parkinson’s disease, remains challenging due to their complex, multifactorial pathogenesis and the limited efficacy of conventional therapies that primarily offer symptomatic relief. The emergence of nanozymes, nanomaterials with intrinsic enzyme-mimicking activities, presents a promising opportunity for multi-targeted intervention. This review comprehensively explores the rational design and therapeutic mechanisms of nanozymes in combating core pathological features of NDs, such as oxidative stress, protein aggregation, and neuroinflammation. We critically assess their remarkable ability to cross the blood–brain barrier and execute synergistic functions, including reactive oxygen species scavenging, misfolded protein degradation, and modulation of microglial activation. Furthermore, we provide a detailed analysis of the application and efficacy of nanozymes across major NDs, alongside a discussion on pharmacokinetics, biosafety, and prevailing challenges. By delineating the structure-enzyme-mimicking activity relationships and cellular-level mechanisms, this review aims to establish a foundational framework for the future development of nanozyme-based neuroprotective strategies, supporting ongoing efforts toward disease-modifying interventions for NDs.

## 1. Introduction

Neurodegenerative diseases (NDs), including Parkinson’s disease (PD), Alzheimer’s disease (AD), amyotrophic lateral sclerosis (ALS), and Huntington’s disease (HD), are a group of pathological conditions characterized by the progressive degeneration of neuronal structure or function, leading to cognitive impairment, motor dysfunction, and even death [1,2]. These have devastating impacts on patients and their families, while effective treatments remain elusive. Although it is not fully understood, accumulating evidence suggested that abnormal protein accumulation and aggregation, oxidative stress, inflammation, and mitochondrial dysfunction play crucial roles in the pathogenesis of NDs [3,4,5]. For instance, PD arises from a vicious cycle where genetic risks trigger α-synuclein (α-syn) aggregation [6,7] and mitochondrial dysfunction, which are then amplified by neuroinflammation and cellular stress [8,9], ultimately leading to the death of dopaminergic neurons and the characteristic motor symptoms [10]. Recent studies have also revealed the roles of lysosomal dysfunction, impaired autophagy, and the pathological spread of α-syn in the gut–brain axis in disease progression, highlighting the systemic and global characteristics of PD pathogenesis [11,12]. Despite significant advances in understanding the pathogenesis of NDs, current therapeutic strategies remain largely palliative, offering only symptomatic relief without halting disease progression [13,14]. The blood–brain barrier (BBB) poses a formidable challenge, limiting the bioavailability of conventional drugs, while the complex, multifactorial nature of NDs—encompassing oxidative stress, protein misfolding, neuroinflammation, and mitochondrial dysfunction—demands interventions capable of simultaneously targeting multiple pathological pathways [15,16]. Existing pharmacological approaches, such as acetylcholinesterase (AChE) inhibitors for AD or DA replacement therapy for PD, fail to address the underlying neurodegeneration, highlighting an urgent need for innovative therapeutic modalities [17,18].

Notably, the emergence of nanotechnology has opened new frontiers in ND treatment, offering precise, multifunctional solutions to longstanding therapeutic hurdles [19,20]. Among these advancements, nanozymes—nanoparticles with enzyme-mimicking catalytic activity—have emerged as a groundbreaking tool, combining the unique physicochemical properties of nanomaterials with the biological functionality of natural enzymes [21]. Notably, pristine nanozymes rarely cross the intact blood–brain barrier efficiently. Nevertheless, rationally engineered nanozymes with surface modification or biomimetic camouflage can achieve limited brain delivery via receptor-mediated transcytosis, and further exert antioxidative, anti-aggregation and anti-inflammatory effects to target core pathological mechanisms of NDs. By scavenging ROS, degrading misfolded proteins, and modulating microglial activation, nanozymes represent a paradigm shift from symptomatic management to disease modification [22,23,24]. Their tunable catalytic activity, coupled with the ability to integrate targeting ligands for site-specific delivery, positions them as a versatile and potent therapeutic platform [25]. Thus, despite the current lack of disease-modifying therapies, nanozymes represent a promising class of candidates for neuroprotection in NDs, although their clinical translation remains to be established [26]. Their multifunctional capabilities and adaptability to diverse pathological contexts make them a promising candidate for next-generation NDs therapies.

In this review, we have focused on the rational design of nanozymes and their cellular-level mechanisms for executing therapeutic interventions against NDs. We delve into the application of nanozymes in combating major NDs—including AD, PD, ALS, and HD—by targeting core pathological features such as oxidative stress, protein aggregation, and neuroinflammation. Furthermore, a comprehensive analysis concerning the pharmacokinetics, safety considerations, existing challenges, and future prospects of nanozyme-based therapeutics has been presented. This review aims to furnish valuable insights and a foundational reference for leveraging nanozymes to advance enzyme-mimicking strategies for the treatment of NDs.

## 2. The Characteristics of Nanozymes

**Figure 1:**
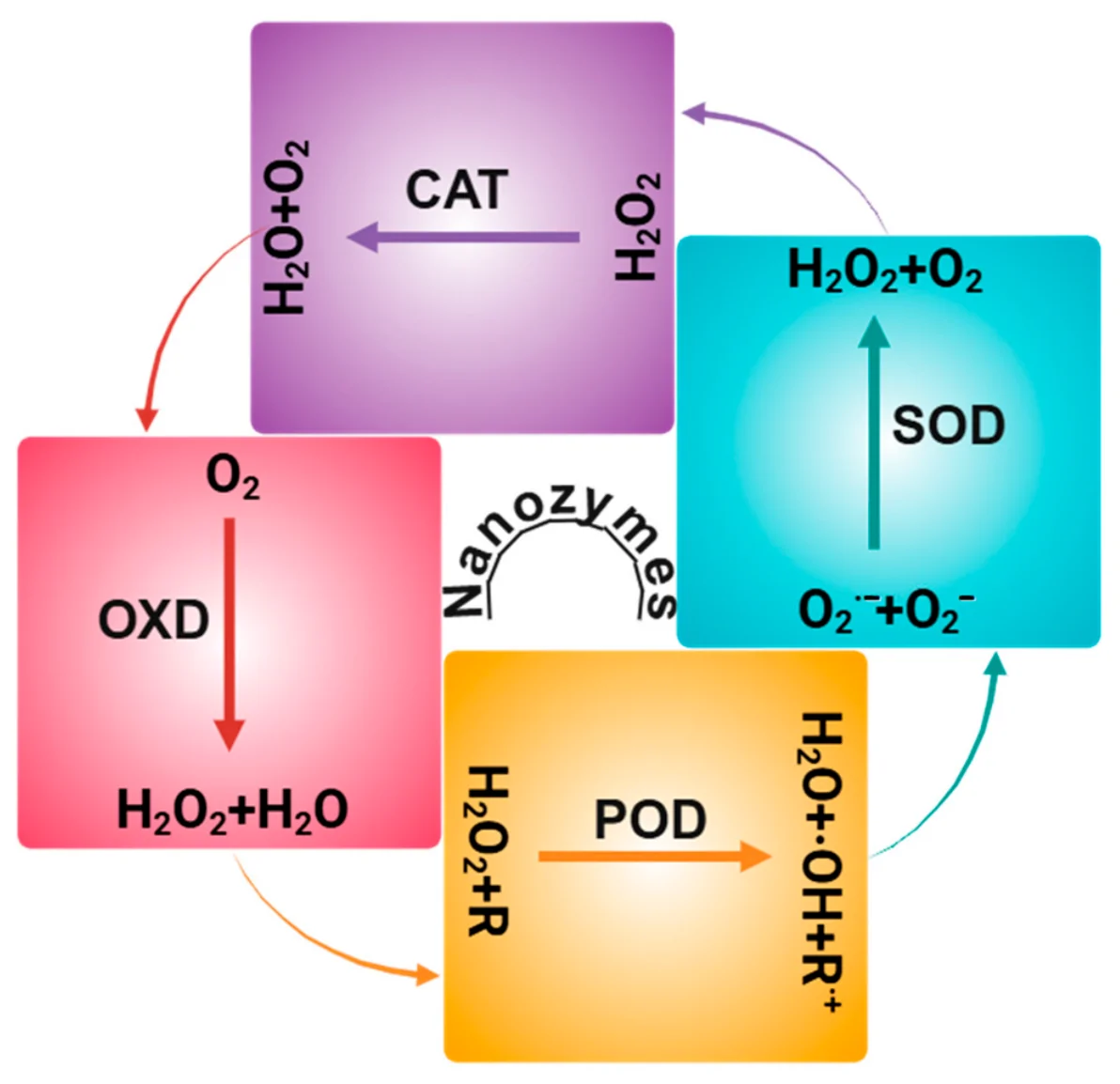
Schematic diagram of the enzyme-like catalytic mechanism of nanozymes.

Nanozymes exhibit diverse catalytic activities—including oxidoreductase-, hydrolase-, lyase-, and isomerase-like functions—making them highly advantageous for various applications [27,28,29]. It is noteworthy that the past decade has witnessed explosive growth in nanozyme research. Among these, oxidoreductase-like nanozymes, which account for over 96% of reported examples and mimic activities such as peroxidase (POD), superoxide dismutase (SOD), oxidase (OXD), glutathione peroxidase (GPx), and catalase (CAT) (Figure 1), represent the most prominent, extensively studied, and consequently well-characterized class [30]. Studies have shown that dysregulation of these enzymatic functions can disrupt cellular redox homeostasis and interfere with essential metabolic pathways—such as those involved in inflammation, oxidative stress, and the metabolism of glucose, lipids, and carbohydrates—which is closely associated with the development of NDs [31,32,33]. For example, Zhang et al. developed an endogenous copper-ion-responsive nanozyme that treats AD by sequestering excess Cu^2+^ in the brain, harnessing NIR-II-enhanced catalysis to inhibit the cGAS-STING pathway, thereby alleviating neuroinflammation and ameliorating cognitive deficits in mice [34]. To address the limited tissue penetration of conventional phototherapy in AD, an amyloid-targeting nanozyme (KD8@N-MCNs) has been developed. This system achieves effective NIR-II photothermal dissociation of Aβ aggregates through the scalp and skull while concurrently alleviating neuroinflammation via its intrinsic antioxidant enzyme activities, ultimately leading to the amelioration of AD pathology in vivo [35]. Therefore, nanozymes provides a biocompatible and non-invasive way to attenuate ND-associated pathology.

It is important to recognize that nanozymes, despite being termed “enzyme mimics,” operate through fundamentally different catalytic mechanisms compared to their natural counterparts. Natural enzymes function as homogeneous catalysts in solution, possessing precisely defined three-dimensional active pockets formed by specific amino acid residues that confer exquisite substrate specificity and high catalytic efficiency under physiological conditions [36]. In contrast, nanozymes typically act as heterogeneous catalysts at solid–liquid interfaces, relying on surface-exposed active sites—such as metal centers, oxygen vacancies, or functional groups—that are structurally ill-defined and distributed non-uniformly [37]. Their catalysis proceeds primarily via surface-mediated redox or electron-transfer pathways rather than the sophisticated acid–base and conformational catalysis characteristic of protein-based enzymes. This mechanistic divergence has profound implications: nanozymes generally exhibit broader substrate tolerance but considerably lower substrate specificity, and their catalytic mechanisms are often less well understood at the atomic level [38].

Surface functionalization strategically modifies nanozymes by introducing specific functional groups or molecules onto their nanomaterial scaffolds. This process enhances key properties—including catalytic efficiency, colloidal stability, substrate selectivity, and biocompatibility—to meet the demands of targeted applications. A prominent research focus in this field is on nanozymes with atomically dispersed metal sites, which offer exceptional catalytic performance and favorable biological characteristics [39]. These well-defined active sites are pivotal for deciphering the structure–activity relationship, a cornerstone for the rational design of high-performance nanozymes. By precisely tuning key structural parameters such as elemental composition, coordination microenvironment, and heteroatom doping, researchers can effectively modulate critical functional metrics like catalytic activity, selectivity, and biological functionality [40,41,42]. In addition, nanozymes can be surface-modified via the deposition of polymer coatings to precisely regulate their surface physicochemical properties. For instance, poly (D,L-lactide-co-glycolide) and poly (lactic-co-glycolic acid) are extensively employed as biomaterial components in nanozyme fabrication, attributed to their excellent biodegradability, favorable biocompatibility, and facile chemical modifiability [43,44,45]. Polydopamine is a well-recognized surface-coating material that exhibits self-polymerization behavior under mild conditions and can spontaneously adhere to the surface of diverse substrates, thereby providing a versatile platform for the construction of multifunctional nanomaterials [46,47,48]. Beyond polymer coatings, nanozymes can also be functionalized with bioactive molecules—including natural enzymes, specific antibodies, or nucleic acids (e.g., DNA)—to endow them with tailored catalytic capabilities or targeted recognition properties [45,49,50]. This functionalization process is typically accomplished through well-established bioconjugation strategies, wherein the bioactive molecules are covalently anchored to the nanozyme surface via site-specific chemical linkages. Collectively, such surface functionalization strategies for nanozymes not only enhance their colloidal stability by suppressing aggregation and improving aqueous dispersibility but also introduce specific functional moieties that contribute to the modulation of catalytic activity or the enhancement of biocompatibility for biological applications.

Typical matrix materials employed for nanozyme fabrication encompass metal nanoparticles (e.g., palladium, platinum, silver, and gold) [51,52,53], metal oxides (such as manganese oxide and cerium oxide) [54], carbon-based nanomaterials (including carbon nanotubes and graphene) [55], and metal–organic frameworks (MOFs) [56,57]. Chen and colleagues have elaborated on the integration of MOFs into the active centers of nanozymes, encompassing OXD-, POD-, CAT-, and SOD-mimetic nanozymes along with their corresponding representative nanozyme systems [58]. Most neuroprotective nanozymes exert catalytic functions via redox-based transformations. For representative oxidoreductase-mimicking activities: in SOD-mimetic catalysis, superoxide anion (O_2_**^−^**) serves as both electron donor and acceptor; one O_2_**^−^** is oxidized to O_2_ and another O_2_**^−^** is reduced to H_2_O_2_, mediated by cyclic electron shuttling over metal active sites [59]. For CAT-mimetic activity, H_2_O_2_ undergoes disproportionation: one H_2_O_2_ molecule is oxidized to O_2_ and another is reduced to H_2_O [58]. In GPx-like reactions, glutathione (GSH) is oxidized to glutathione disulfide (GSSG), accompanied by H_2_O_2_ reduction to H_2_O. For POD-like activity, H_2_O_2_ is reduced to H_2_O while organic co-substrates are oxidized. In neurodegenerative pathological contexts, major physiological reaction partners are pathological reactive oxygen/nitrogen species, glutathione, and other endogenous biological reductant. For metal-containing nanozymes, reversible valence- state transitions of metal centers constitute the core catalytic cycle, including well-known redox pairs: Ce^3+^/Ce^4+^, Mn^2+^/Mn^3+^/Mn^4+^, Fe^2+^/Fe^3+^, and Cu^+^/Cu^2+^ [58]. These metal ions are not functionally identical. Their inherent redox potential, alongside local coordination microenvironment, jointly dictates catalytic selectivity, activity, and biosafety in brain tissue [58,60].

The selection of specific materials is contingent upon application-specific requirements and targeted catalytic performance metrics. As a representative illustration, researchers have constructed a nanozyme through two synergistic modification approaches: encapsulation of L-arginine and ultrasmall gold nanoparticles within hyaluronic acid-based matrices, coupled with the immobilization of Cu_1.6_O nanoparticles onto phosphorus-doped graphitic carbon nitride nanosheets [61]. Notably, this rationally engineered nanozyme exhibits five distinct enzyme-mimetic activities, specifically POD-, CAT-, SOD-, nitric oxide synthase-, and glucose oxidase-mimetic activities [61]. Grasping the catalytic mechanism of the target enzyme is fundamental to the rational design of nanozymes with desired enzyme-mimetic activities. Through in-depth investigation of the reaction pathway and precise identification of rate-limiting steps as well as key intermediate-involved steps, researchers are enabled to tailor nanozymes that can recapitulate these critical reaction processes and thereby mediate the identical catalytic reaction with comparable efficiency.

Overall, the rational design of nanozymes with tailored enzyme-mimetic activities entails a multi-step workflow, encompassing the screening and selection of suitable nanomaterial scaffolds, precise surface functionalization to modulate physicochemical properties, in-depth elucidation of intrinsic catalytic mechanisms, systematic optimization of structural and functional parameters, and ultimately validation of their performance in target-specific application scenarios. This rapidly evolving research field exhibits immense potential for advancing the development of innovative catalytic systems, which are expected to feature enhanced catalytic efficiency, superior stability, and expanded applicability across diverse fields.

## 3. The Pathogenesis of NDs

**Figure 2:**
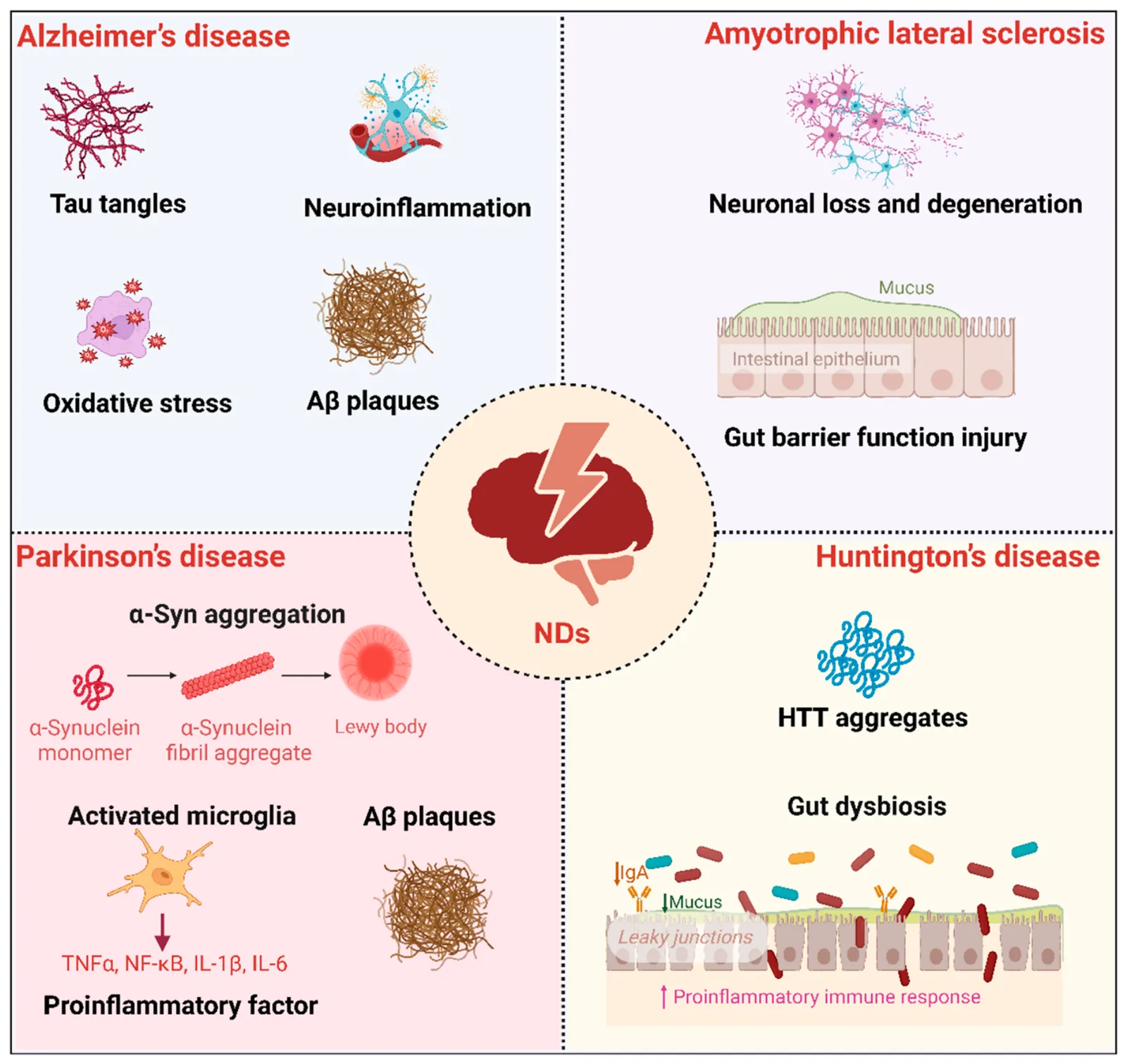
The core pathological hallmarks and shared mechanisms across major NDs. Created in BioRender. Y.W. (2026); https://BioRender.com.

The exact mechanisms underlying neurodegeneration and the onset and progression of these diseases remain controversial, while recent studies have offered important insights into their pathogenesis (Figure 2). One prevailing hypothesis is that the NDs are caused by the accumulation of abnormal protein aggregates, including amyloid β (Aβ) in AD, huntingtin (HTT) in HD, and α-syn in PD, among others [62]. These proteins are produced in all cells, including neurons. They could misfold and form insoluble aggregates that might disrupt normal cellular functions and lead to neuronal death [63,64]. The accumulation of these protein aggregates also activated microglia, the immune cells that reside in the brain, and cause chronic inflammation, which exacerbates neurodegeneration and further contributes to the disease process [65].

Oxidative stress is marked by the disparity between the antioxidant defense system and the generation of ROS, resulting in cellular damage and dysfunction, and has been implicated as a significant factor to the pathophysiology of several NDs. For example, in AD, ROS production is elevated due to the accumulation of Aβ and tau protein, which induce mitochondrial dysfunction and eventually lead to neuronal cell death [66]. Similarly, in PD, ROS are generated by the dysfunction of the mitochondrial respiratory chain, which promotes the aggregation of α-syn and subsequent neuroinflammation [67]. The activation of nicotinamide adenine dinucleotide phosphate oxidase and the accumulation of misfolded proteins increased ROS production, which induces neuronal dysfunction and degeneration in ALS [68]. Several studies have demonstrated the roles of antioxidants in the management of NDs. For instance, the administration of vitamins C and E has been shown to reduce oxidative stress and improve cognitive function in AD patients [69].

Notably, genetic susceptibility and environmental factors also perform key roles in the developing and progression of NDs. For example, mutations in genes associated with protein homeostasis, such as presenilin 1 (PSEN1), amyloid precursor protein (APP), and PSEN2, have been linked to early onset AD [70]. The occurrence of HD has been associated with mutations in the *HTT* gene, whereas mutations in the *SOD1* gene have been linked to ALS [71,72]. Meanwhile, environmental factors such as exposure to toxins and pollutants, traumatic brain injury, and lifestyle factors have been linked to increased risk for NDs [73].

The involvement of neuroinflammation performs a crucial role in the development of NDs [74]. It is characterized by the activation of immune cells and the secretion of various chemical mediators, including cytokines, chemokines, and ROS. In NDs, neuronal damage leads to the release of these chemical mediators, which further activate immune cells and trigger neuroinflammation. Neuroinflammation exacerbates neuronal damage and contributes to the progression of NDs through several mechanisms. It disrupts normal cellular processes, impairs the ability of neurons to repair themselves, and activates cell death pathways. Additionally, neuroinflammation can damage the BBB, allowing harmful substances to enter the brain and accelerate the development of NDs [75]. Overall, the interaction between neuronal damage and neuroinflammation creates a vicious cycle that drives the progression of NDs.

It has become increasingly evident that neurodegenerative disorders also affect peripheral systems, including the gastrointestinal (GI) tract [76]. The GI tract is innervated by the ENS, which consists of the myenteric plexus and submucosal plexus. The myenteric plexus, located between the circular and longitudinal muscle layers of the GI tract, plays a crucial role in regulating GI motility and coordinating peristaltic movements [77]. In neurodegenerative disorders, the myenteric plexus and its nerve supply can degenerate, leading to impaired GI function. This dysfunction in the GI tract can manifest as various GI symptoms, including constipation, delayed gastric emptying, and dysphagia. These symptoms are commonly observed in patients with PD [78]. The degeneration of the myenteric plexus and disruption of neural signaling can result in decreased GI motility and altered gut transit time. Furthermore, the alterations in the GI tract due to neurodegenerative disorders can also impact the gut microbiota, which play a crucial role in maintaining GI health and overall well-being. The disrupted GI function in neurodegenerative disorders can lead to changes in the gut environment, such as altered pH levels, decreased mucus production, and impaired intestinal barrier function [79]. These changes can create an imbalance in the gut microbiota composition, known as dysbiosis, which has been observed in various neurodegenerative disorders, such as AD and PD [80]. The degeneration of the myenteric plexus and disruption of neural signaling can lead to GI dysfunction, which, in turn, can alter the gut microbiota [81]. Understanding these complex interactions between the nervous system, GI function, and the gut microbiota is crucial for developing potential therapeutic strategies for neurodegenerative disorders [82]. In conclusion, although the exact mechanisms underlying neurodegeneration and the onset and progression of NDs remain unclear, recent studies have shed light on the significant contributions of protein misfolding, autophagy deficiency, and genetic and environmental factors to their pathogenesis. Understanding these mechanisms could pave the way for the development of effective therapeutic strategies to mitigate or even reverse the disease process.

## 4. Nanozyme Design Principles for Brain Applications for Increasing the Permeability of BBB

NDs represent a clinically and pathologically diverse class of neurological conditions characterized by the progressive degeneration and irreversible loss of post-mitotic neurons within the central or peripheral nervous systems [83]. This pathological cascade leads to the structural and functional disintegration of neural networks, ultimately manifesting as debilitating impairments across multiple domains, including cognitive, behavioral, sensory, and motor functions [84]. The development of nanozymes for NDs requires meticulous engineering to address the unique challenges of the brain microenvironment, including the restrictive BBB, complex neuroinflammatory signaling, and the need for precise catalytic activity within neural tissues (Table 1). Effective nanozyme design integrate multiple functional components to achieve targeted delivery, controlled catalytic behavior, and biocompatibility while mitigating off-target effects [85]. The BBB is a highly selective neurovascular interface formed by tight-junction-linked brain capillary endothelial cells in intimate association with basement membrane, pericytes, astrocytes, microglia and neurons; this ensemble maintains central nervous system (CNS) homeostasis by excluding blood-borne toxins and most therapeutics—only lipophilic small molecules traverse passively [86,87]. Consequently, the BBB constitutes the principal pharmacological obstacle in AD and PD. Recent advances in nanomedicine have exploited surface-functionalized nanoparticles—liposomes, microemulsions, micelles—and targeting ligands such as cell-penetrating peptides, transferrin and lactoferrin to engage BBB receptors, thereby enabling receptor-mediated transcytosis and markedly enhancing brain-targeted drug delivery.

### 4.1. Coating with Cell Membranes

Cell membranes, as native constituents of living organisms, inherently impart excellent biocompatibility to nanocarriers. Relative to synthetic membrane coatings, cell-membrane camouflage leverages the endogenous repertoire of proteins, peptides and enzymes to afford markedly enhanced diagnostic and therapeutic performance in disease contexts [88]. Consequently, membrane-coated nanoparticles exhibit prolonged systemic circulation, augmented active targeting and elevated cellular uptake [89]. For example, Ma et al. engineered a Prussian-blue biomimetic nanozyme doped with cerium and cloaked in a neutrophil-derived membrane, a strategy that simultaneously heightened BBB permeability, sharpened lesion-selective targeting, and concomitantly curtailed nonspecific accumulation, while in vivo fluorescence imaging further confirmed the robust BBB traversal and focal enrichment of NM@PB-Ce within the intended cerebral region [90]. NM@PB-Ce exhibits multi-enzyme mimetic activity (CAT, SOD, POD), effectively scavenges ROS, suppresses NLRP3 inflammasome activation, and reduces Aβ aggregation. It efficiently crosses the BBB and accumulates in AD-affected regions. In an Aβ-induced AD mouse model, NM@PB-Ce treatment improved cognitive function, reduced neuroinflammation (GFAP, Iba-1), decreased NLRP3/caspase-1 and p-tau levels, and enhanced dendritic spine density, demonstrating its potential as a targeted AD therapeutic [90]. Similarly, cloaking CuxO nanozyme with erythrocyte membranes harvested from 3xTg-AD mice and subsequently functionalized with the Aβ-directed peptide KLVFF (CuxO@EM-K) markedly prolongs their colloidal stability and systemic half-life, enabling the augmented sequestration of peripheral Aβ and its efficient shuttling to the liver for metabolic clearance. Within this construct, the Aβ peptide motif serves as a high-affinity ligand that, in concert with the membrane’s intrinsic tropism, selectively captures circulating Aβ, whereas the erythrocyte-derived envelope simultaneously suppresses non-specific protein-corona formation, thereby preserving the nanozyme’s targeting integrity [91].

### 4.2. Designing Specific Targeted Ligands

In addition, the rational design of ligands that specifically target receptors highly expressed on the brain endothelium—such as low-density lipoprotein receptor-related protein (LRP1) or lactoferrin receptors (LfR)—can significantly enhance the delivery efficiency of engineered nanozymes across the BBB through receptor-mediated transcytosis, thereby enabling precise brain-targeted therapy while minimizing systemic off-target effects [92,93]. For example, angiopep-2 functionalization, achieved through conjugation to a biodegradable dendrigraft poly-L-lysine (DGL)-based vector via a polyethyleneglycol (PEG) spacer, significantly enhances BBB penetration and targeted gene delivery by leveraging its high-affinity binding to the LRP1 abundantly expressed on brain endothelial cells, thereby facilitating receptor-mediated transcytosis and promoting robust neuroprotective effects in a chronic model of PD [94]. Similarly, angiopep-2 functionalization, in conjunction with PEG surface modification, significantly enhances the BBB permeability of edaravone-loaded ceria nanozymes (E-A/P-CeO_2_) by leveraging the high binding affinity of angiopep-2 for the LRP1, thereby facilitating efficient receptor-mediated transcytosis across intact BBB and enabling targeted antioxidant delivery and ROS scavenging within ischemic brain tissues for synergistic neuroprotection and stroke therapy [95]. In addition, lactoferrin (Lf) functionalization, achieved through covalent conjugation to a β-cyclodextrin (β-CD)-based nanocarrier via a heterobifunctional PEG spacer (NHS-PEG-MAL), significantly enhances BBB permeability and brain-targeted delivery by leveraging the high-affinity binding of Lf to LfR abundantly expressed on cerebral endothelial cells, thereby facilitating robust receptor-mediated transcytosis and resulting in a seven-fold increase in the area under the concentration–time curve (AUC_0–2h_) of the encapsulated cargo in the brain compared to the free drug [96]. Li et al. reported that Lf functionalization, through surface conjugation to Au-Bi_2_Se_3_ nanodots, significantly enhances BBB permeability by leveraging its high-affinity interaction with LfR, thereby facilitating robust receptor-mediated transcytosis and enabling efficient antioxidant and mitochondrial protection within the substantia nigra pars compacta for PD therapy [97]. Taken together, functionalization with cell membrane-derived coatings or specific targeting ligands—such as angiopep-2 or lactoferrin—markedly enhances the BBB permeability of nanozymes through efficient receptor-mediated transcytosis, thereby substantially improving their targeted delivery efficiency and therapeutic utility in the treatment of NDs.

### 4.3. Disputes and Limitations

The evidence for brain entry is heterogeneous and often indirect, for example, many studies rely on in vivo fluorescence imaging or whole-brain homogenates, which cannot separate nanozymes that have crossed the endothelium from those bound to, or retained within, the cerebral vasculature; capillary-depletion assays or cellular-resolution imaging are rarely reported. Moreover, some designs were validated in models in which barrier integrity is itself altered, as in the ischemic stroke model used for angiopep-2-modified ceria [95], or relied on a transient permeability enhancer (borneol [98]) or an external trigger (NIR-induced opening [99,100]). In these cases the measured penetration may overestimate what can be expected from the largely intact barrier of early-stage NDs. One example, CuxO@EM-K [91], does not enter the brain and acts as a peripheral Aβ sink; it represents a different therapeutic concept rather than a BBB-crossing strategy. In addition, scalability and systemic toxicity are linked. Membrane cloaking [90,91] and EV-based systems [99] prolong circulation and reduce non-specific uptake, but they depend on cell or vesicle sources that are difficult to standardise, and disease-model-derived membranes used in proof-of-concept work cannot be transferred directly to patients. Longer circulation also increases exposure of the liver and spleen to redox-active metals, which is relevant for chronic dosing. Ligand-based approaches (angiopep-2 [95], lactoferrin [97]) are chemically more reproducible, but they act on receptors shared with endogenous ligands, whose expression may change with disease stage, and ligand density affects protein-corona formation and thereby uptake by the mononuclear phagocyte system Trigger-dependent or local strategies such as NIR irradiation [100] or microneedle delivery [101] avoid some of these issues but add tissue-depth limits, heat-related risk or invasiveness. No single strategy therefore dominates; the most suitable choice is likely to depend on disease stage, dosing frequency and whether a focal (e.g., nigral) or diffuse (e.g., cortical) target is intended. Meanwhile, the route of transport may determine the catalytic outcome. Receptor-mediated transcytosis and subsequent microglial or neuronal uptake route nanoparticles through endosomal and lysosomal compartments. For nanozymes with peroxidase-like activity, which is generally favoured at acidic pH, accumulation in these compartments could shift catalysis from ROS scavenging towards ROS generation [102]. This coupling between delivery route and catalytic function is rarely tested directly. Collectively, BBB-crossing nanozyme strategies remain limited by indirect and context-dependent evidence of brain entry, unresolved scalability–toxicity trade-offs, and the risk that intracellular routing redirects catalytic activity, so no single approach dominates and the optimal choice depends on disease stage, dosing, and target location.

In summary, the strategic design of brain-targeted nanozymes for enhanced permeability primarily revolves around two pivotal principles: biomimetic camouflage and specific ligand engineering. Coating nanozymes with natural cell membranes (e.g., from neutrophils or erythrocytes) confers superior biocompatibility, prolongs systemic circulation, and leverages endogenous membrane proteins to augment active targeting and cellular uptake. Concurrently, functionalization with specific targeting ligands, such as angiopep-2 or lactoferrin, facilitates highly efficient receptor-mediated transcytosis across the BBB by engaging highly expressed receptors (e.g., LRP1, LfR) on brain endothelial cells. These synergistic strategies collectively ensure precise brain delivery, minimize off-target effects, and form the cornerstone of effective nanozyme-based therapeutics for neurological disorders.

Future research trajectories will focus on developing next-generation “smart” nanozymes with dynamically responsive capabilities. The frontier lies in engineering nanozymes whose catalytic activity and targeting functions can be selectively activated or modulated by specific pathological stimuli within the brain microenvironment, such as pH, enzymes, or ROS gradients. This will maximize therapeutic precision while minimizing healthy tissue exposure. Furthermore, the integration of multi-modal functionalities-combining real-time diagnostic imaging, synergistic therapeutic actions (e.g., antioxidant, anti-aggregation), and immunomodulation-into a single platform represents a pivotal direction for achieving theranostic applications. Ultimately, overcoming the translational challenges of scalable manufacturing, long-term biosafety, and personalized design will be crucial to bridge the gap from preclinical promise to clinical reality in treating NDs.

**Table 1:** Representative BBB-crossing strategies for nanozymes: evidence and limitation.

Platform	BBB Strategy	Brain-Delivery Evidence as Reported	Limitation	Reference
NM@PB-Ce	Neutrophil-membrane cloak	In vivo fluorescence imaging; AD mouse model	Imaging is semi-quantitative; parenchymal vs. vessel-bound signal not resolved; membrane sourcing and batch variability	[90]
CuxO@EM-K	Erythrocyte-membrane cloak + KLVFF	Does not cross the BBB; peripheral Aβ sink	Mechanism is peripheral; membranes from 3xTg-AD mice are not clinically transferable	[91]
E-A/P-CeO_2_	Angiopep-2/LRP1 transcytosis	Ischemic stroke model	Not an ND model; the BBB is disrupted after ischemia, which may overestimate penetration in NDs	[95]
Au-Bi_2_Se_3_-Lf	Lactoferrin/LfR transcytosis	PD model; substantia nigra enrichment	Possible competition with endogenous ligand and altered receptor expression; long-term fate of Bi/Se unknown	[97]
Pd@PEG@Bor	Borneol modification	AD mouse model; behavioural rescue	Borneol is a non-selective permeability enhancer; safety of repeated barrier modulation unclear	[98]
Res-Pt@EVs-RVG	RVG-engineered EVs + Pt photothermal effect	AD mouse model	EV isolation is hard to standardise and scale; photothermal contribution	[99]
MXenzyme	NIR-II-triggered BBB opening	AD mouse model	Requires external NIR; tissue-depth limit in humans; heat-related tissue risk	[100]
MFeI microneedle	Local parenchymal delivery	PD model; substantia nigra and striatum	Invasive; Fe loading in an iron-rich nigra needs long-term assessment	[101]

## 5. The Application of Nanozyme in NDs

### 5.1. AD

AD, a progressive neurodegenerative disorder, has emerged as a critical global public health concern, driving research efforts toward early diagnosis and etiology-targeted therapies [103]. In clinical practice, approved pharmacological treatments primarily aim to alleviate symptoms and slow disease progression, yet they do not address the underlying pathological mechanisms [104]. The lack of specificity of these conventional drugs often leads to undesirable side effects, further complicating treatment outcomes [103,105]. Moreover, current diagnostic approaches rely heavily on clinical symptoms and neuroimaging, which lack sensitivity for early-stage detection, thereby limiting opportunities for timely intervention [106]. Nanozymes have garnered significant research interest for their potential in the diagnosis and treatment of AD, owing to their high stability, tunable catalytic activity, and superior specificity, which collectively enable novel diagnostic and therapeutic strategies [107]. For instance, they can be engineered to mimic natural enzymes involved in the clearance of toxic Aβ aggregates or to serve as sensitive probes for detecting AD-related biomarkers at early stages. Thus, nanozymes represent an emerging platform that may overcome the limitations of current methods, providing enhanced accuracy in diagnosis and targeted molecular intervention for AD.

#### 5.1.1. The Application of Nanozymes in the Diagnosis of AD

In the diagnosis of AD, nanozymes have emerged as powerful tools for detecting key biomarkers with high sensitivity and specificity, addressing the limitations of conventional diagnostic methods such as ELISA, which is often invasive, time-consuming, and lacks early detection capability [108]. Key AD biomarkers include AChE, tau protein, Aβ, glutathione (GSH), cysteine, DA, hydrogen peroxide, and hydrogen sulfide, which are present in cerebrospinal fluid or blood and can reflect pathological changes years before clinical symptoms appear [109,110]. Nanozymes, with their enzyme-mimicking activities, enable precise in vitro and in vivo detection of these biomarkers [111]. For instance, Li et al. developed a dual-mode fluorescence-photothermal assay for sensitive detection of AChE activity by leveraging the oxidase-mimetic property of Cu-BTC nanozymes to catalyze o-phenylenediamine (oPD) into fluorescent oligomers and photothermal J-aggregated polymer nanoparticles. This method quantifies AChE activity through thiocholine-induced suppression of Cu-BTC activity, achieving low detection limits (0.03 U·L^−1^ for fluorescence, 0.05 U·L^−1^ for photothermal) and reliable determination in human serum via complementary dual-signal readout [112]. For Aβ detection, Wang et al. constructed a self-powered nanozyme imaging strategy in which Pt nanozymes catalyze the deoxygenation of non-fluorescent resazurin into highly fluorescent resorufin, enabling real-time fluorescent tagging of Aβ aggregates. The resulting fluorescent resorufin binds specifically to Aβ via synergistic π-stacking and hydrogen-bonding interactions, thereby conferring fluorescence to the aggregates and allowing their detection through both in vitro and in vivo fluorescence microscopy [113]. Notably, a multifunctional nanocomposite targeting hyperphosphorylated tau was constructed through controlled assembly of ultrasmall ceria nanocrystals (CeNCs) and iron oxide nanocrystals (IONCs) onto mesoporous silica nanoparticles (MSNs). This nanozyme-integrated platform enables bimodal MRI/PET imaging via surface-immobilized IONCs and Ga, offering a novel diagnostic strategy for AD. Furthermore, by incorporating methylene blue (MB) as a tau aggregation inhibitor, the nanocomposite exhibits enhanced synergistic therapeutic efficacy, outperforming individual components in both diagnosis and treatment of AD [114].

In addition, nanozyme-based sensing strategies have been developed for the highly selective detection of GSH and DA. The PdFe-decorated graphdiyne nanozyme (PdFe/GDY) exhibits POD-like activity and catalyzes the oxidation of TMB in the presence of H_2_O_2_ to form a blue product. The introduction of GSH reduces this product, resulting in a decrease in absorbance at 652 nm, which enables sensitive and selective detection even in complex biological samples [115]. An amorphous imidazolium–copper nanozyme (I–Cu) mimics both catecholase and laccase activities and directly oxidizes DA to produce a distinct black polydopamine product, offering high specificity and strong anti-interference capability against common metabolites such as glucose and lactic acid, with a detection limit of 0.412 mM [116]. Taken together, nanozymes have emerged as pivotal diagnostic tools for AD, enabling high-sensitivity and high-specificity detection of a panel of key biomarkers such as AChE, Aβ, and tau through their multi-enzyme mimetic activities, while the integration of multimodal imaging and multi-signal output strategies significantly enhances the accuracy and reliability of early diagnosis.

#### 5.1.2. The Application of Nanozymes in the Treatment of AD

Nanozymes have emerged as a novel and promising therapeutic frontier. These artificial enzymes offer unique advantages over their natural counterparts, such as enhanced stability, tunable catalytic activity, and the capacity for multi-functional integration. By catalytically eliminating ROS, inhibiting the aggregation of pathogenic proteins, and modulating the inflammatory microenvironment, nanozymes target multiple core pathological pathways of AD simultaneously [117]. This part aims to comprehensively explore the current landscape of nanozyme-based strategies for AD treatment for harnessing these innovative nanotherapeutic agents to combat this devastating disorder. Nanozymes show great potential for multi-targeted, synergistic interventions in the treatment of AD.

A primary application lies in mitigating oxidative stress, a central driver of AD pathogenesis. Various nanozymes, such as cerium dioxide (CeO_2_) nanoparticles utilizing a Ce^3+^/Ce^4+^ redox switch, palladium hydride (PdH) for selective hydroxyl radical scavenging, and selenium quantum dots (SeQDs), mimic endogenous antioxidant enzymes like SOD and CAT to neutralize ROS, thereby preserving mitochondrial function and neuronal health [118]. Beyond merely scavenging ROS, the strategic design of nanozymes aims to concurrently address the intertwined pathophysiological hallmarks of NDs. Jia et al. developed an octahedral palladium nanozyme (Pd@PEG@Bor) modified with borneol to enhance BBB penetrability [98]. This construct exhibited potent dual enzyme-mimetic activities, functioning as both a POD and SOD, which cooperatively mitigated cytotoxic oxidative stress and preserved mitochondrial membrane potential. Crucially, this robust antioxidative efficacy translated into tangible therapeutic benefits by attenuating the formation and deposition of Aβ aggregates. Consequently, Pd@PEG@Bor administration was demonstrated to significantly ameliorate cognitive deficits in AD mouse models, underscoring the potential of multi-functional nanozymes to disrupt the vicious cycle between oxidative stress and proteinopathy in AD [98]. Similarly, Bai et al. developed a brain-targeted nanozyme platform by co-loading multi-enzyme mimicking platinum nanoparticles and the natural antioxidant resveratrol into rabies virus glycoprotein (RVG)-functionalized extracellular vesicles. This biomimetic system, Res-Pt@EVs-RVG, synergistically mitigated AD pathogenesis by enhancing BBB penetration-aided by a Pt-induced photothermal effect-efficiently scavenging ROS, ameliorating mitochondrial dysfunction, suppressing neuroinflammation, and ultimately rescuing memory deficits in AD mice [99]. Fu et al. generated ROS-scavenging Pd-In that mitigates AD-like phenotypes, including cognitive impairment, excessive ROS accumulation, Aβ deposition, neuroinflammation, and neuronal loss [119].

Escalating evidence underscores neuroinflammation as a critical pathogenic driver in AD, transitioning from a mere consequence to a key perpetrator in the disease’s self-sustaining cycle [120,121]. This chronic inflammatory state, primarily orchestrated by activated microglia and astrocytes, is fueled by the pathological accumulation of Aβ plaques and neurofibrillary tangles [122,123]. Consequently, targeting neuroinflammation has emerged as a pivotal therapeutic strategy for interrupting the vicious cycle of AD pathogenesis [124]. Nanozyme utility extends beyond conventional antioxidant functions; by scavenging reactive oxygen and nitrogen species that act as key inflammatory signaling mediators, nanozymes can effectively suppress the overactivation of microglia and shift their phenotype from a pro-inflammatory (M1) to an anti-inflammatory (M2) state [125]. This direct modulation of the neuroinflammatory microenvironment by nanozyme not only alleviates neuronal damage but also contributes to reducing the burden of pathogenic protein aggregates, positioning nanozyme-mediated immunomodulation as a compelling approach for disease-modifying AD therapy [126]. For example, a neutrophil-membrane-camouflaged mesoporous Prussian blue nanozyme (MPBzyme@NCM) was developed, which achieves noninvasive active targeting to the inflamed brain vasculature and, upon microglial uptake, orchestrates a comprehensive therapeutic cascade including microglial polarization toward the M2 phenotype, inhibition of neutrophil recruitment, suppression of neuronal apoptosis, and promotion of neurogenesis [127]. Similarly, Ren et al. designed mitochondria-targeted TPP-MoS2 QD nanozymes to mitigate AD pathology by crossing the BBB, targeting mitochondria, and polarizing microglia toward the anti-inflammatory M2 phenotype, thereby resolving neuroinflammation and eliminating Aβ aggregates, thus demonstrating a therapeutic pathway distinct from direct affinity-based strategies [128]. Mn_3_O_4_ nanozymes exert anti-inflammatory effects in a 5 × FAD mouse model by reprogramming microglia via the TLR4/NOX2 pathway. This intervention establishes a critical temporal link wherein the polarization of microglia to an M2 phenotype leads to the sequential resolution of neuroinflammation and, ultimately, the clearance of Aβ pathology and recovery of cognitive function [129].

Transition metals such as iron, manganese, copper, and zinc play a paradoxical role in brain health: while serving as essential cofactors for fundamental physiological processes, their homeostatic imbalance—whether deficiency or excess—can trigger multiple pathogenic cascades [60,130]. These include oxidative stress, ferroptosis, cuproptosis, and cellular senescence, which collectively drive neuroinflammation and neuronal death, thereby constituting a central mechanism in the pathogenesis of NDs [131,132]. Consequently, rectifying this cerebral metallostasis presents a compelling strategy for disease modification. In this context, nanozymes offer a novel and sophisticated platform to intervene in this complex process. Their utility extends beyond simple antioxidant mimicry; by exhibiting CAT- and SOD-like activities, they can directly scavenge metal-induced ROS. More profoundly, certain designer nanozymes can function as metal chelators or catalytic metal centers themselves, selectively sequestering or modulating redox-active metal ions to prevent Aβ aggregation and disrupt the vicious cycle of oxidative stress and inflammation. This dual capacity to simultaneously regulate the cerebral microenvironment and correct underlying ionic imbalances positions nanozyme technology as a promising frontier for developing multi-targeted therapeutic interventions against AD. For example, a two-dimensional niobium carbide (Nb_2_C) MXene-based nano-chelator and nanozyme (MXenzyme) was developed by Du et al. to address copper ion (Cu^2+^) dyshomeostasis and oxidative stress in AD. The designed MXenzyme not only effectively chelates pathogenic Cu^2+^ to suppress Aβ peptide aggregation but also exhibits potent antioxidant activity to scavenge ROS, with its mechanism elucidated via computational simulation. Furthermore, its benign photothermal effect enhances BBB permeability under near-infrared irradiation, overcoming a major delivery challenge and presenting a comprehensive neuroprotective strategy against AD [100]. Similarly, a synergistic, H_2_O_2_-responsive nano-platform was engineered by combining a metal chelator and CeO_2_NPs for AD therapy. This system, which pioneers the use of CeO_2_NPs as both a capping and antioxidant agent, leverages the dual functions to inhibit Aβ aggregation and scavenge ROS, thereby offering enhanced neuroprotection compared to individual components [133]. In addition, to address metal ion-induced protein aggregation in neurological diseases, Cui et al. developed nanoparticle conjugates of the Cu(I) chelator D-penicillamine using cleavable disulfide or thioether linkages. These conjugates demonstrated excellent colloidal stability under physiological conditions and selectively released the active chelator under reducing conditions, mimicking the intracellular environment. The released D-penicillamine effectively resolubilized copper-induced Aβ(1–42) aggregates, demonstrating the potential of this nanoparticle platform for targeted brain delivery to combat Aβ accumulation and metal dyshomeostasis [134]. Despite these encouraging pre-clinical findings, several caveats should be noted. Most existing studies employ relatively short-term animal experiments, which cannot fully recapitulate the slow, heterogeneous progression of human Alzheimer’s disease. Inconsistent therapeutic performance can arise from differences in nanozyme composition, surface modification and animal model selection. Furthermore, head-to-head comparative investigations and long-term in vivo biosafety assessments are still largely absent, restricting direct extrapolation toward clinical scenarios.

### 5.2. PD

PD, the most common neurodegenerative disorder, poses a substantial and growing challenge to global healthcare systems, underscoring an urgent need for advanced management strategies [135,136]. In current clinical practice, the cornerstone of pharmacological treatment, such as levodopa, primarily focuses on replenishing DA to alleviate motor symptoms, yet fails to halt the underlying neurodegeneration or address the multifaceted pathology, including α-syn aggregation and oxidative stress [137]. The chronic administration of these therapies is often complicated by diminishing efficacy and debilitating side effects, such as levodopa-induced dyskinesia [138,139]. Furthermore, conventional diagnosis predominantly relies on the manifestation of characteristic motor symptoms (e.g., tremor, bradykinesia), which typically emerge only after significant dopaminergic neuron loss has already occurred, thereby missing a critical window for early intervention [140,141]. For instance, they can be engineered to catalytically scavenge ROS implicated in dopaminergic neuronal death, inhibit metal-induced α-syn fibrillation, or function as contrast agents for sensitive neuroimaging. Consequently, nanozymes represent a transformative platform with the potential to overcome the diagnostic sensitivity barriers and therapeutic shortcomings of existing PD management paradigms.

#### 5.2.1. The Application of Nanozymes in the Diagnosis of PD

The timely and accurate diagnosis of PD remains a significant challenge in clinical neurology, as the characteristic motor symptoms emerge only after substantial and irreversible loss of dopaminergic neurons in the substantia nigra. Current diagnostic protocols, which rely heavily on clinical observation and neuroimaging, often lack the sensitivity for detecting pre-symptomatic or early-stage disease, thereby missing critical windows for early intervention. Nanozymes have surfaced as powerful tools poised to revolutionize PD diagnostics. Their unique advantage lies in their ability to be engineered as multifunctional biosensing platforms. For instance, nanozymes with POD-like activity can catalyze colorimetric reactions for the highly sensitive detection of PD-specific biomarkers, such as aberrant α-syn oligomers in biofluids or subtle changes in DA metabolites [142]. Zhu et al. developed cubic Prussian blue nanoparticle-supported MoS_2_ nanocomposites (MoS_2_-CPBNPs) as robust POD-mimicking nanozymes for the colorimetric detection of DA. The detection mechanism relies on the inhibitory effect of DA on the nanozyme-catalyzed oxidation of TMB, enabling a sensitive and label-free assay with a wide linear range (0–300 μM) and a low detection limit (0.09 μM). The high accuracy demonstrated in practical analyses underscores the potential of this sensor for biomedical diagnostics in PD [143]. A wash-free colorimetric sensor was developed based on a chitosan-stabilized Prussian blue nanozyme (CS/PBNPs) with intrinsic POD activity, integrated with a DA-specific aptamer. This platform operates via a radical-mediated TMB oxidation mechanism, demonstrating high selectivity and sensitivity for DA detection with a wide linear range (0.25–100 μM) and a low detection limit (0.16 μM), outperforming a reference AuNP-based aptasensor. The excellent recovery in human serum, coupled with the elimination of washing steps, confirms its reliability and practical potential for streamlined clinical diagnostics [144]. Similarly, a chemically etched Co-Fe Prussian blue analog (PBA) nanozyme with superior POD-like activity, outperforms natural horseradish POD due to enhanced electronic and structural properties that facilitate substrate binding. Leveraging this high catalytic efficiency, the etched PBA enabled a sensitive colorimetric platform for detecting H_2_O_2_ and DA via a TMB oxidation-mediated strategy, achieving detection limits as low as 0.083 μM and 0.05 μM, respectively. The successful application in real-sample analysis confirms its significant potential in biosensing and diagnostic fields [145]. Zhao et al. engineered a sensitive fluorescence platform for DA monitoring using Fe-MIL-88 MOFs to catalyze the H_2_O_2_-OPD reaction system. The sensor operates through a metal-enhanced catalytic mechanism where DA promotes ·OH production, leading to significantly amplified fluorescence output. With excellent analytical performance (LOD: 46 nM) and successful application in complex matrices, this strategy offers a reliable approach for DA quantification in pharmaceutical and clinical settings [146]. In summary, by offering a versatile, sensitive, and specific approach to biomarker detection, nanozymes hold immense promise for shifting the PD diagnostic paradigm from one based on late-stage clinical signs to one grounded in early molecular evidence, ultimately facilitating timely and personalized therapeutic strategies.

#### 5.2.2. The Application of Nanozymes in the Treatment of PD

Current therapeutic strategies primarily focus on symptomatic management through DA replacement therapy, yet they fail to halt disease progression and often lead to debilitating side effects with long-term use. The complex pathogenesis of PD involves multiple interconnected pathways, including mitochondrial dysfunction, oxidative stress, neuroinflammation, and the accumulation of α-syn aggregates, creating a self-perpetuating cycle of neuronal degeneration [1,147].

The pivotal role of chronic neuroinflammation, driven largely by the persistent activation of microglia, in the pathogenesis of PD has established it as a critical therapeutic target. Nanozymes, with their programmable catalytic activities and superior biocompatibility, have emerged as a novel class of anti-neuroinflammatory agents capable of intervening in this destructive process. Their therapeutic mechanism is multifaceted: they can directly scavenge ROS that serve as key inflammatory signaling mediators, thereby disrupting the cycle of oxidative stress and inflammation [101]. More profoundly, certain nanozymes have been engineered to actively polarize microglia from the pro-inflammatory M1 phenotype, which releases cytotoxic cytokines, to the neuroprotective M2 phenotype, which promotes tissue repair and resolution of inflammation [148]. This phenotypic switch is often achieved through the specific inhibition of inflammatory signaling pathways, such as the TLR4/NF-κB or NLRP3 inflammasome axes [149,150]. By simultaneously quenching oxidative stress and reprogramming microglial function, nanozymes effectively reduce the release of inflammatory factors (e.g., TNF-α, IL-1β), protect dopaminergic neurons from inflammation-induced apoptosis and ferroptosis, and ultimately ameliorate motor deficits and pathological progression in PD models, presenting a promising disease-modifying strategy [151]. For example, Jiang et al. developed chiral nanozyme-embedded metal–organic frameworks (Ptzyme@L/D-ZIF) as anti-neuroinflammatory agents for PD treatment. The D-enantiomer (Ptzyme@D-ZIF) demonstrated superior brain accumulation due to longer plasma half-life and enhanced BBB transendocytosis, leading to remarkable therapeutic efficacy by alleviating neuroinflammation and inhibiting neuronal apoptosis and ferroptosis. These findings elucidate the critical role of chirality in nanomedicine distribution and efficacy, providing a novel strategic platform for anti-PD drug development [151]. Liu et al. developed a novel microneedle (MN) system for in situ brain delivery of mitochondrion-targeted single-atom nanozymes (MFeI) to overcome multiple biological barriers in PD treatment. The MFeI-loaded MNs enabled direct parenchymal delivery and enhanced drug accumulation in key pathological regions like the substantia nigra and striatum, where the nanozymes effectively scavenged ROS and alleviated neuroinflammation. This targeted approach demonstrated superior therapeutic efficacy in ameliorating behavioral deficits and pathological symptoms compared to conventional intravenous administration [101]. Similarly, Li et al. developed a mannitol-functionalized liposomal nanozyme system (MOF@Man Liposome) by encapsulating a Zr-FeP metal–organic framework for targeted PD therapy. The constructed nanozyme effectively crosses the BBB via mannitol-mediated transport and demonstrates potent anti-neuroinflammatory effects by specifically inhibiting NLRP3 inflammasome formation and assembly. Through transcriptomic and biochemical analyses, the system was shown to mitigate glial activation, reduce neuroinflammation, and alleviate PD pathological symptoms by restoring mitochondrial function and suppressing inflammasome signaling pathways [152]. The rational design of a biomimetic nanozyme (R-NM@Fe-Ic)—integrating Icariside II-Fe^3+^ coordination and RVG29-neutrophil membrane coating—yielded a targeted PD therapeutic with dual enzyme-like activities, effectively combating pathology through ferroptosis inhibition (via lipid peroxidation suppression/GPX4 upregulation), α-syn reduction, and mitophagy promotion, leading to superior in vivo outcomes over L-DOPA in BBB penetration, brain targeting, and motor/cognitive recovery, thus offering a novel combinatorial neuroprotection platform [153].

Oxidative stress represents a pivotal pathological mechanism in PD, characterized by excessive ROS generation and compromised antioxidant defense systems, ultimately leading to dopaminergic neuronal degeneration. Nanozymes can simultaneously mimic key antioxidant enzymes enabling them to efficiently scavenge various ROS and maintain redox homeostasis in the nigrostriatal pathway. By neutralizing superoxide anions, hydrogen peroxide, and hydroxyl radicals, nanozymes protect mitochondrial function, prevent lipid peroxidation, and reduce protein oxidation, thereby preserving neuronal integrity. For instance, Prussian blue nanozyme (PBzyme) serves as an effective pyroptosis inhibitor for PD therapy by specifically targeting the NLRP3 inflammasome pathway [149]. Both in vitro and in vivo experiments demonstrated that PBzyme exerts significant neuroprotective effects through scavenging ROS, suppressing NLRP3 inflammasome activation and caspase-1 cleavage, and consequently inhibiting gasdermin D-mediated pyroptosis in microglia. The treatment alleviated motor deficits, rescued dopaminergic neurons, and reduced neuroinflammation in MPTP-induced PD models, thereby proposing a promising therapeutic strategy for NDs [149]. Biodegradable tannic acid–iron complex nanoparticles (BSA/Fe-TA NPs) with atomically dispersed iron sites and multienzyme activities were developed for comprehensive PD therapy. These nanoparticles efficiently cross the BBB, accumulate in neural cells, and simultaneously alleviate oxidative stress by scavenging reactive oxygen/nitrogen species while promoting microglial polarization toward the anti-inflammatory M2 phenotype. The system demonstrated significant therapeutic efficacy in PD mice without adverse effects and featured controllable degradation facilitated by clinical iron-chelating ligands, thereby providing a safe and effective strategy for disrupting the oxidative stress–neuroinflammation cycle in neurodegeneration [154]. Collectively, these nanozyme systems protect mitochondrial function, inhibit lipid peroxidation, and reduce protein oxidation, effectively disrupting the vicious cycle between oxidative stress and neuroinflammation in PD, thereby opening new avenues for developing disease-modifying therapies against neurodegenerative disorders.

Nanozymes demonstrate multifunctional therapeutic potential in countering α-syn pathology, a defining neuropathological feature of PD [37,155]. Beyond their established antioxidative properties, these catalytic nanomaterials directly interfere with α-Syn aggregation dynamics through dual mechanisms: they effectively disrupt preformed fibrillar assemblies while preventing the initial nucleation and co-aggregation of monomeric and oligomeric species [26,156]. This dual-action approach positions nanozyme technology as a promising strategy for modifying the fundamental protein pathway underlying PD progression. Liu et al. address the critical challenge of inhibiting prion-like α-syn propagation in PD by developing PtCu nanoalloys as antioxidant nanozymes. The nanozymes effectively blocked α-syn pathology transmission between neurons and suppressed its spreading in PFF-injected mouse models through efficient ROS scavenging. This pioneering work provides the first demonstration of nanozymes capable of inhibiting prion-like protein spreading, establishing a novel therapeutic concept for PD and related proteinopathies [157]. The neuroprotective mechanism of cerium oxide nanoparticles (CeO_2_NPs) against PD’s pathology was elucidated using a yeast model expressing human α-syn. The nanoparticles conferred dose-dependent protection against α-syn-induced toxicity by inhibiting cytoplasmic aggregate formation, redirecting α-syn to the plasma membrane, and ameliorating mitochondrial dysfunction and ROS production. Notably, in vitro binding assays confirmed direct adsorption of α-syn on the surface of CeO_2_NPs, thereby demonstrating a dual mechanism combining radical scavenging with direct protein interaction [158]. Collectively, they demonstrate significant multi-target therapeutic advantages in the treatment of PD by synergistically ameliorating three core pathological processes: oxidative stress, neuroinflammation, and α-syn aggregation. Looking forward, the application potential of nanozymes in PD therapy is expected to be further unlocked through the rational design of intelligently responsive nanozymes and continuous optimization of their biocompatibility. By integrating targeting peptide modification and biomimetic membrane coating technologies, the next generation of nanozymes is anticipated to achieve precise lesion-targeted delivery and on-demand catalytic activity, thereby opening new avenues for the development of disease-modifying therapies capable of simultaneously intervening in multiple pathological pathways.

Beyond canonical ROS scavenging, nanozymes intervene in PD pathology through several mechanistically distinct routes. α-Syn aggregation and propagation are inhibited both by direct surface adsorption—CeO_2_ nanoparticles bind α-syn and redirect it to the plasma membrane, suppressing cytoplasmic aggregation—and by ROS scavenging, as PtCu nanoalloys block neuron-to-neuron α-syn transmission and prion-like spreading [157,158]. Microglial M1 → M2 switching is driven primarily by suppression of ROS-mediated TLR4/NOX2 signaling, as exemplified by Mn_3_O_4_ nanozymes [129]. TLR4/NLRP3 modulation occurs through ROS scavenging and inhibition of upstream oxidative signaling; PBzyme suppresses NLRP3 inflammasome and caspase-1 activation, inhibiting gasdermin D-mediated pyroptosis and rescuing dopaminergic neurons in MPTP-induced PD models [149]. Metal dyshomeostasis is corrected by chelation or sequestration of redox-active ions: R-NM@Fe-Ic integrates Fe^3+^-Icariside II coordination as both catalytic center and iron chelator, restoring iron homeostasis, suppressing ferroptosis, and inhibiting α-syn aggregation [153]. Collectively, nanozymes intervene in PD through a coordinated network of surface adsorption, ROS-mediated signaling modulation, metal chelation, and immune reprogramming rather than a single catalytic reaction. Although multiple nanozyme platforms produce desirable neuroprotective phenotypes in PD animal models, several limitations persist. Current in vivo assessments mostly focus on short-term behavioral and pathological readouts. Few works systematically evaluate long-term risks including metal-ion leakage, chronic neuroinflammation triggered by nanomaterials, and real efficiency of α-syn transmission inhibition under more physiologically relevant conditions. Standardized benchmark assays are urgently needed to compare different nanozyme candidates across laboratories.

### 5.3. ALS

ALS, a progressive and fatal neurodegenerative disorder characterized by selective loss of upper and lower motor neurons, presents a formidable challenge to global healthcare systems due to its devastating clinical course and limited treatment options [159,160]. In current clinical management, pharmacological interventions such as riluzole and edaravone offer modest symptomatic relief or modestly slow disease progression but fundamentally fail to halt the underlying neurodegenerative processes or address the multifactorial pathology, including TDP-43 proteinopathy, persistent oxidative stress, and dysregulated neuroinflammation [160,161,162]. Furthermore, chronic treatment is often constrained by limited efficacy and potential side effects, while diagnosis predominantly relies on the manifestation of unequivocal motor symptoms—such as muscle weakness, spasticity, and bulbar impairments—which typically emerge only after significant motor neuron loss has occurred, thereby missing the critical window for early therapeutic intervention [163,164,165]. For instance, Chen and colleagues developed a dual-mode colorimetric/photothermal sensing platform based on an iodide-synergized covalent porphyrin-triazine framework nanozyme (Zn-CTF/I) for ultrasensitive AChE detection, where iodide coordination optimized the zinc active sites’ electronic structure to dramatically enhance peroxidase-like activity—achieving an exceptional detection limit of 0.003 U L^−1^ (tenfold lower than controls) with high reliability in serum samples, thereby offering a robust analytical tool for early neurodegenerative disease diagnosis [166]. Singh et al. demonstrate for the first time that a cerium vanadate (CeVO_4_) nanozyme can functionally substitute for both SOD1/SOD2 in neuronal cells, even under specific gene silencing conditions [167]. The nanozyme effectively regulated superoxide levels, prevented mitochondrial damage and depolarization, and restored physiological expression of anti-apoptotic Bcl-2 family proteins in SOD-deficient cells. Furthermore, it significantly improved cellular ATP levels under oxidative stress, establishing its potential as a complete functional replacement for natural enzymes in therapeutic contexts [167]. By integrating ultra-sensitive biomarker detection capabilities with multifunctional therapeutic interventions—ranging from ROS scavenging and mitochondrial protection to functional enzyme replacement—nanozymes offer a comprehensive strategy to overcome the limitations of current diagnostic and therapeutic modalities. Their ability to simultaneously target multiple pathological mechanisms, including TDP-43 proteinopathy, oxidative stress, and neuroinflammation, positions these nanostructures as promising candidates for developing early intervention therapies and personalized treatment regimens for this devastating neurodegenerative disorder.

### 5.4. HD

HD is an autosomal dominant neurodegenerative disorder caused by a CAG repeat expansion in the HTT gene, posing complex clinical management challenges and a significant burden on global healthcare systems [168,169]. Current therapeutic approaches primarily focus on symptomatic management—such as tetrabenazine for chorea control and antipsychotics for psychiatric symptoms—yet fail to modify disease progression or target the underlying pathogenic mechanisms, including mutant huntingtin (mHTT) aggregation, mitochondrial dysfunction, and persistent neuroinflammation [170,171,172]. Moreover, the diagnosis of HD still heavily relies on genetic testing and the onset of overt motor symptoms, which typically emerge only after substantial striatal neuronal loss has occurred, thereby limiting the window for early intervention [173,174]. For instance, Adhikari and colleagues developed a citrate-functionalized manganese oxide nanozyme (C-Mn_3_O_4_ NP) that effectively mimics GPx activity under physiological conditions and integrates into cellular metabolic networks to scavenge hydrogen peroxide. Combining computational and experimental approaches, the team elucidated the nanozyme’s catalytic mechanism and demonstrated its therapeutic efficacy in a HD animal model. This work introduces a paradigm-shifting strategy for utilizing biocompatible nanozymes as functional elements within cellular enzymatic networks to combat neurodegenerative disorders [175]. In addition, to address key pathological mechanisms of HD, a novel amino-nanozyme was engineered based on boehmite nanoparticles (BNPs) functionalized with a tetra-azapyridinophane ligand (L1). The L1 ligand itself forms a Cu^2+^ complex with potent SOD activity. When grafted onto the BNPs, the resulting L1-BNP conjugate not only exhibits enhanced mitochondrial ROS (mitoROS) scavenging properties but also demonstrates the ability to disaggregate mutant HTT deposits in cellular models, showcasing multifunctional therapeutic potential [176]. Nanozymes address critical pathological hallmarks of HD, offering an integrated approach to scavenge mitoROS and resolve mHTT aggregates.

## 6. In Vivo Fate of Nanozymes in ND

**Figure 3:**
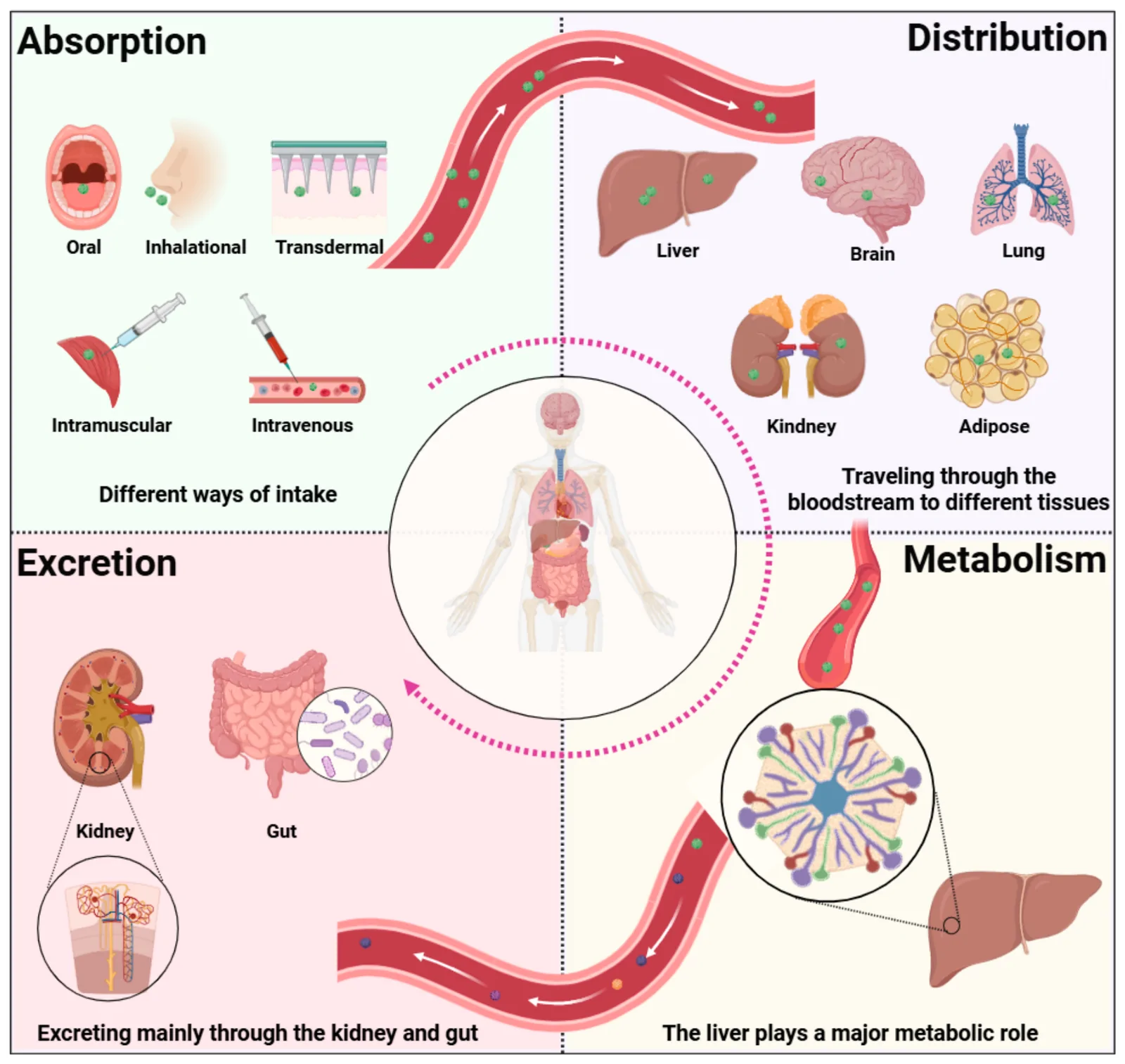
The schematic diagram illustrating the processes of absorption, distribution, metabolism, and excretion of nanozymes in the body. Created in BioRender. Y.W. (2026); https://BioRender.com.

For nanozymes intended to treat neurodegenerative disorders (NDs), the in vivo journey begins with administration—chiefly intravenous injection, with intranasal and oral routes also under exploration [149,177,178,179], and is defined by a fundamental difference from peripheral nanomedicines: the BBB, rather than leaky tumor vasculature, constitutes the dominant biological barrier, and the diseased brain offers no equivalent of the enhanced permeability and retention effect. Absorption and cellular internalization therefore hinge on the nanozyme’s intrinsic physicochemical properties, such as size, surface charge, and functionalization, which determine its interactions with endothelial surfaces and the mucosal/epithelial barriers it encounters [180,181]. Upon reaching the target cell, internalization proceeds chiefly by endocytosis, with the specific pathway (clathrin-mediated, caveolae-mediated, or macropinocytosis) dictated by the nanozyme’s characteristics and the recipient cell type [182,183,184,185]. This is of direct therapeutic relevance in the CNS, where the effector populations differ markedly in uptake behavior: because neuroinflammation is propagated largely through microglial and astrocytic activation, nanozymes must be engineered to engage neurons, microglia, and astrocytes in a cell-type-specific manner rather than being internalized indiscriminately [186]. A comprehensive understanding of these absorption and internalization mechanisms is therefore pivotal for the rational design of next-generation nanozymes with optimized bioavailability, precise subcellular targeting, and enhanced therapeutic efficacy (Figure 3).

Following systemic administration, nanozymes enter the circulation and are distributed via the bloodstream and lymphatic system [187], a process shaped by blood flow, tissue vascular permeability, and the tropism of the mononuclear phagocyte system (MPS) [44,188] together with the nanozyme’s size, surface charge, and hydrophobicity [189]. A pivotal early event is the rapid formation of a protein corona that masks the original synthetic identity and redefines subsequent biological fate [187,190]. For brain delivery, this corona is doubly consequential: it can obscure engineered targeting ligands, but it can also be exploited. For example, ApoE-enriched coronas engage low-density-lipoprotein-receptor-related protein trafficking and have been co-opted to ferry nanoparticles across the BBB [186]. Because the intact BBB lacks the leaky vasculature that passively accumulates nanomedicines in tumors, distribution of the nanoplatforms highlighted throughout this review must be actively engineered rather than left to passive diffusion. Cell-membrane cloaking has emerged as a leading strategy to this end: a neutrophil-membrane-coated, cerium-doped Prussian blue nanozyme (NM@PB-Ce) traverses the BBB in a co-culture model and accumulates specifically in Aβ-bearing regions of AD mice [90], while neutrophil-membrane-coated mesoporous Prussian blue MOF nanozymes exploit the natural neutrophil–inflamed-endothelium interaction to reach the ischemic brain after stroke [186]. Erythrocyte- and microglia-membrane-coated platforms likewise leverage self-derived membrane ligands to cross the BBB and home to neuroinflammatory lesions. These membrane-cloaked systems, among the specific therapeutic nanoplatforms discussed earlier in this review, are governed not only by particle size but by the membrane’s own protein landscape, which determines both BBB traversal and lesion tropism. After reaching the target tissue, subcellular localization endosomal, lysosomal, cytoplasmic, or mitochondrial, is dictated by the delivery strategy [191,192]. Rational design must therefore optimize size and surface chemistry to modulate corona formation and stealth properties while integrating targeting motifs and stimuli-responsive elements; for CNS delivery this task is especially exacting, as both the BBB and the blood–cerebrospinal-fluid barrier must be negotiated.

The metabolism of nanozymes in vivo is intimately tied to their chemical stability, constituent materials, and the biological milieu they encounter. Biodegradable nanozymes can be hydrolyzed or degraded within the acidic lysosomal environment or by biological enzymes; metal-based nanozymes may undergo valence changes and slow ion release that influence catalytic activity and raise toxicity concerns [30]. Iron oxide nanozymes can degrade and release iron ions, whereas cerium oxide nanozymes cycle between Ce^3+^ and Ce^4+^ on their surface [193,194]. These considerations acquire special significance in the brain. Iron released within the CNS can feed the neuronal iron dyshomeostasis and ferroptosis that contribute to PD and AD pathology, whereas cerium, for which the body lacks a dedicated metabolic processing pathway, may accumulate in brain and reticuloendothelial tissues if not cleared [195]. Moreover, in the complex microenvironments of the diseased brain, catalytically active sites can be obscured by adsorbed biomolecules or undergo irreversible chemical modification, leading to reduced or lost enzyme-mimetic activity, an effect directly documented for platinum nanozymes, whose activity is suppressed by the serum corona yet restored upon lysosomal corona degradation [195]. A deep understanding of these in vivo metabolic pathways is therefore crucial for evaluating long-term safety and efficacy, and for NDs it must explicitly include how degradation products interact with neurons and glial cells.

Excretion represents the terminal phase of the nanozyme’s in vivo journey and proceeds chiefly through renal and hepatobiliary pathways, governed by size, surface charge, coating, and morphology. Renal excretion is generally restricted to small, stable nanozymes or biodegradable fragments with hydrodynamic diameters below ~5–10 nm, enabling passage through the glomerular filtration barrier into urine; larger species are cleared predominantly via the feces [196], including unabsorbed orally administered nanozymes and those undergoing hepatobiliary excretion [197]. Secondary clearance involves MPS macrophages transporting nanozymes to the liver or spleen, lymphatic capture and degradation within lymph nodes, and rarely exhalation of volatile or gas-generating species [198]. Two considerations are peculiar to CNS nanozymes. First, although peripheral clearance can be efficient, the brain parenchyma and glymphatic system clear nanoparticles slowly, so the very platforms engineered to cross the BBB membrane-coated Prussian blue, ceria, or single-atom systems, may persist within the CNS, where chronic microglial exposure risks sustained neuroinflammation. Second, incomplete clearance and accumulation in MPS organs such as the liver and spleen pose long-term toxicity risks [199]. Timely and complete clearance is therefore of paramount importance for translational safety, and a systematic understanding of nanozyme excretion, particularly across the BBB, is an essential prerequisite for advancing clinical translation.

## 7. Clinical Translation Challenges

Despite the considerable promise demonstrated by nanozymes in preclinical studies for neurodegenerative diseases, their clinical translation encounters substantial hurdles. Key challenges include unclear biosafety profiles, with unresolved issues regarding long-term toxicity, immunogenicity, and metabolic clearance pathways; the complex pathological microenvironment, which complicates the precise regulation of catalytic activity and stability; and the translational gap between animal models and humans, as existing models fail to fully recapitulate the chronic progression and heterogeneity of human neurodegenerative conditions. These fundamental issues collectively impede the transition of nanozymes from laboratory research to clinical application.

### 7.1. Biosafety and Long-Term Toxicity: The Primary Hurdle for Clinical Application

The most significant challenge in the clinical translation of nanozymes is the uncertainty regarding their biosafety and long-term toxicity [200]. Although designed to mimic the high catalytic efficiency of natural enzymes, their intrinsic nature as nanomaterials introduces unique risks. Firstly, immunogenicity and inflammatory responses are critical concerns [201,202]. Nanoparticles are readily recognized and phagocytosed by the immune system upon administration, potentially triggering sustained neuroinflammation [203]. Given that neuroinflammation is a core pathway exacerbating the pathology of many NDs, this could paradoxically worsen the condition. Secondly, the long-term in vivo fate of nanozymes remains largely unknown. Prolonged retention within the CNS could induce secondary neuronal damage through mechanisms such as oxidative stress, lysosomal dysfunction, or autophagic disruption [204]. Finally, the enzymatic properties of nanozymes themselves can be a double-edged sword. For instance, nanozymes with peroxidase-like activity, if present in excess or in specific microenvironments, might catalyze the production of excessive ROS, thereby aggravating oxidative damage [102]. Therefore, a systematic evaluation of their in vivo distribution, metabolism, accumulation, and long-term biocompatibility is an absolute prerequisite for clinical advancement.

### 7.2. Efficient BBB Penetration and Lesion-Specific Targeting: The Major Bottleneck in Precise Delivery

A prerequisite for effectively treating NDs is the ability of therapeutic agents to cross the BBB and selectively accumulate at the site of pathology. However, efficient traversal of the BBB represents a formidable challenge. While surface functionalization with targeting ligands can enhance penetration, the efficiency and specificity of this approach can vary significantly between individuals and disease stages, potentially introducing new immunogenic risks. More critically, achieving specific targeting within the brain parenchyma is even more difficult. Pathological lesions are often diffusely distributed and intermingled with healthy neurons. A key question remains: how can nanozymes be designed to specifically bind to pathological agents without affecting normal cellular function? The lack of this cellular or molecular precision results in most of the administered dose being distributed in healthy brain tissue, which not only reduces therapeutic efficacy but also increases the risk of off-target toxicity [205]. The development of “smart” nanozymes that can respond to the pathological microenvironment is a crucial direction for future research to achieve effective and safe delivery.

### 7.3. Scalable Manufacturing and Quality Control: The Translational Gap from Lab to Industry

The synthesis of nanozymes in laboratory settings often involves complex procedures with poor reproducibility and low yield, failing to meet the stringent requirements for large-scale, reproducible, and cost-effective production necessary for clinical and commercial application [206]. The catalytic activity of nanozymes is highly dependent on their physical and chemical properties. Even minor fluctuations in synthesis parameters can lead to significant batch-to-batch variations, directly impacting their safety and efficacy profile. Furthermore, robust quality control standards are yet to be established. For such novel biologics, it is imperative to define critical quality attributes and establish standardized assays to ensure that every batch is consistent and meets predefined specifications. Bridging this gap requires deep collaboration across disciplines to establish a robust and controllable manufacturing process.

### 7.4. The Lack of a Robust Translational Pathway: Uncharted Territory from Preclinical to Clinical Evaluation

Translating nanozymes into clinical use requires a robust evaluative framework that can accurately predict their efficacy and safety in humans, which is currently underdeveloped. Preclinical models for NDs are limited in profound ways: APP/PS1 and other amyloid models, MPTP- or α-synuclein-based PD models, and Aβ-oligomer-induced models do not capture the chronic, heterogeneous, multi-decade course of human neurodegeneration, and the acute oxidative-stress windows in which nanozymes show benefit may not reflect human disease [195]. There is also a critical lack of consensus on biological and clinical endpoints for evaluating nanozyme efficacy. In animal models, does clearance of a certain percentage of amyloid plaques necessarily translate to cognitive improvement? Notably, NM@PB-Ce produced behavioral improvement alongside reduced plaque and inflammatory markers [90] yet the dissociation between plaque clearance and cognition observed in clinical trials of amyloid-directed antibodies cautions against assuming such a linkage. In human trials, which biomarkers, amyloid or α-synuclein PET, cerebrospinal-fluid markers, redox biomarkers, or functional measures, should serve as primary endpoints? These unresolved questions highlight the urgent need to develop more predictive models and to define standardized efficacy metrics across different stages of clinical development to successfully guide the translation of nanozymes.

## 8. Conclusions and Future Perspectives

In summary, nanozymes represent a promising therapeutic platform with considerable potential for addressing the multifaceted pathophysiology of NDs. Their capacity to mimic natural enzyme activities, combined with the tunable physicochemical properties of nanomaterials, enables them to concurrently target the core pathological mechanisms, including oxidative stress, protein misfolding, and neuroinflammation, which have largely evaded conventional monotherapies. Crucially, rationally designed nanozymes that can traverse the BBB and execute synergistic functions, such as scavenging ROS and degrading aberrant protein aggregates, offer a powerful means of achieving disease modification rather than mere symptomatic relief.

Looking forward, several key avenues warrant focused exploration to advance nanozyme therapeutics toward clinical translation. First, the long-term biosafety, biodegradation, and clearance profiles of nanozymes must be systematically evaluated in relevant disease models, with particular attention to their potential accumulation in off-target organs. Second, the rational design of nanozymes must evolve towards greater sophistication. This includes engineering stimuli-responsive “smart” nanozymes that are activated specifically within the pathological microenvironment of the diseased brain, thereby maximizing therapeutic efficacy while minimizing off-target effects. Third, the integration of multi-enzyme mimicking cascades within a single nanostructure could more effectively restore the disrupted metabolic networks in NDs. Finally, the combination of nanozyme-mediated neuroprotection with other modalities, such as drug delivery or neuromodulation, may offer a comprehensive therapeutic strategy. By tackling these challenges through interdisciplinary collaboration, nanozyme-based interventions are poised to transition from a promising concept to a tangible reality, ultimately offering hope for halting the progression of these currently intractable disorders.

## Data Availability

No new data were created or analysed in this study. Data sharing is not applicable to this article.
